# Unpleasant, but a Duty, Nevertheless

**Published:** 1890-06

**Authors:** 


F. E. DANIEL, M. D., EDITOR AND PUBLISHER.

THIS JOURNAL, by unanimous vote of the members, was made the Official Organ of the Austin District Medical Society.

COLLABORATORS:

*Dr. R. M. Swearingen, Austin.*

*Prof. B. E. Hadra, M. D., Galveston.*

*Prof. Geo. Cupples, M. D., San Antonio.*

*Dr. T. C. Osborn, Cleburne, Texas.*

*Dr E. J. Doering, Chicago.*

*Dr. E. J. Beall, Fort Worth.*

*Dr Odo Betz, Germany.*

*Dr. J. W. McLaughlin, Austin.*

*Dr. T. J. Tyner, Austin.*

*Prof. J. F. Y. Paine, M. D., Galveston.*

*Dr. R. H. L. Bibb, Mexico.*

*Dr. T. J. Bennett. Austin.*

*Dr. Bat Smith, Wharton.*

*Dr. E. Meierhof. New York.*

*Prof. W. B. Rogers, M. D., Memphis, Tenn.*

### UNPLEASANT, BUT A DUTY, NEVERTHELESS.

If we understand the mission of the Medical JOURNAL it is to mould professional thought, and reflect medical sentiment; and, pointing out evils and errors, suggest remedies and reforms; to stimulate a healthy interest in professional progress, and to incite to a high standard of moral and professional character.

This JOURNAL at its inception, declared its aim to be "the advancement, elevation and purification of the medical profession of Texas." We have to date, endeavored to carry out this idea, and have "hewed to the line," regardless of where the chips have fallen.

That there are moral and professional sores on the body medical—goes without saying. Whether they be amenable to journalistic treatment remains to be seen; certain it is, to attack quackery in the profession is not so popular as to make war on the outside quacks.

It is too often the case that journals are owned by non-professional publishers; their object is to make money; hence they must "run with the hare and hold with the hounds;" must not offend anybody for fear of losing patronage. There is no remedy for this; but where the journal is owned and controlled by a professional man—and is sincerely conducted in the interest of legitimate medicine, if that journal does not speak out, and attack the evils known to exist in the profession—who shall do so?

We are in position to *tell the truth.* We shall indulge in no personalities, and intend to insult no one; but if in our criticisms of the regular profession; in pointing out evils and indicating needed reforms in our ranks—the cap should fit any—they must wear it. While we prize the patronage so generously bestowed on our efforts, we will not truckle to hold it, or to secure additional.

In our next will be an article in pursuance of this idea which may make some wince; and we say now that no names be-called, the principle, or the representative of an idea or class is meant.

Medical associations should represent the highest professional and moral element. We do not want every body, who, by virtue of the possession of a diploma is recognized as a member of the regular profession. The aim is, or should be, to enroll every worthy and honorable physician in the State, as the first step in the winnowing process of separating the wheat from the chaff, and of consolidating the strength of the profession, looking to exerting such influence with the legislature as will secure the needed protection both for the public and the profession; but, at the same time, the societies being as much social as scientific, in their character, and at their meetings members are wont to introduce the ladies of their households, a diligent circumspection should govern their action in the matter of admitting applicants to membership. That there are men in certain State Medical Associations, in full fellowship, of questionable moral character, nay, even, of known depravity, and whom a gentleman would hesitate to introduce in his family, is well known. It is a lamentable fact that there are some in the State Association who cannot join the societies where they live.

This evil, we presume, must be born; at least, so long as they behave themselves with due decency; but in future the closest scrutiny into moral character and professional integrity should be exercised with regard to applications for membership. Applicants are taken in, sometimes, with the mistaken notion that to throw the restraint of the code around them will be to reform them. As well try to change the spots of the leopard; a quack will be a quack, in or out of the medical associations; and having already some of that stamp in the State organizations, care should be exercised to prevent the admittance of others:

